# Comparison of Propylthiouracil vs Methimazole for Thyroid Storm in Critically Ill Patients

**DOI:** 10.1001/jamanetworkopen.2023.8655

**Published:** 2023-04-17

**Authors:** Sun Y. Lee, Katherine L. Modzelewski, Anica C. Law, Allan J. Walkey, Elizabeth N. Pearce, Nicholas A. Bosch

**Affiliations:** 1 Department of Medicine, Section of Endocrinology, Diabetes, Nutrition & Weight Management, Boston University Chobanian & Avedisian School of Medicine, Boston, Massachusetts; 2 Department of Medicine, The Pulmonary Center, Boston University Chobanian & Avedisian School of Medicine, Boston, Massachusetts

## Abstract

**Importance:**

Thyroid storm is the most severe form of thyrotoxicosis, with high mortality, and is treated with propylthiouracil and methimazole. Some guidelines recommend propylthiouracil over methimazole, although the difference in outcomes associated with each treatment is unclear.

**Objective:**

To compare outcomes associated with use of propylthiouracil vs methimazole for the treatment of thyroid storm.

**Design, Setting, and Participants:**

This comparative effectiveness study comprised a large, multicenter, US-based cohort from the Premier Healthcare Database between January 1, 2016, and December 31, 2020. It included 1383 adult patients admitted to intensive or intermediate care units with a diagnosis of thyroid storm per *International Statistical Classification of Diseases and Related Health Problems, Tenth Revision* codes and treated with either propylthiouracil or methimazole. Analyses were conducted from July 2022 to February 2023.

**Exposure:**

Patients received either propylthiouracil or methimazole for treatment of thyroid storm. Exposure was assigned based on the initial thionamide administered.

**Main Outcomes and Measures:**

The primary outcome was the adjusted risk difference of in-hospital death or discharge to hospice between patients treated with propylthiouracil and those treated with methimazole, assessed by targeted maximum likelihood estimation.

**Results:**

A total of 1383 patients (656 [47.4%] treated with propylthiouracil; mean [SD] age, 45 [16] years; 473 women [72.1%]; and 727 [52.6%] treated with methimazole; mean [SD] age, 45 [16] years; 520 women [71.5%]) were included in the study. The standardized mean difference for age was 0.056, and the standardized mean difference for sex was 0.013. The primary composite outcome occurred in 7.4% of of patients (102 of 1383; 95% CI, 6.0%-8.8%). A total of 8.5% (56 of 656; 95% CI, 6.4%-10.7%) of patients who initiated propylthiouracil and 6.3% (46 of 727; 95% CI, 4.6%-8.1%) who initiated methimazole died in the hospital (adjusted risk difference, 0.6% [95% CI, −1.8% to 3.0%]; *P* = .64). There were no significant differences in duration of organ support, total hospitalization costs, or rates of adverse events between the 2 treatment groups.

**Conclusion and Relevance:**

In this comparative effectiveness study of a multicenter cohort of adult patients with thyroid storm, no significant differences were found in mortality or adverse events in patients who were treated with propylthiouracil or methimazole. Thus, current guidelines recommending propylthiouracil over methimazole for treatment of thyroid storm may merit reevaluation.

## Key Points

**Question:**

What is the difference in mortality after treatment of thyroid storm with propylthiouracil vs methimazole?

**Findings:**

In this comparative effectiveness study using a large, multicenter, US-based cohort that included 1383 adults admitted to intensive care units or intermediate care units with thyroid storm, no significant difference in mortality was found between patients treated with propylthiouracil or patients treated with methimazole.

**Meaning:**

This study suggests that current guidelines recommending propylthiouracil over methimazole for treatment of thyroid storm may merit reevaluation.

## Teaser

This comparative effectiveness study uses a large, multicenter, US-based cohort to compare outcomes associated with use of propylthiouracil vs methimazole for the treatment of thyroid storm.

## Introduction

Thyroid storm is characterized by thyrotoxicosis that leads to life-threatening acute end-organ damage, including neurologic dysfunction, cardiogenic shock, hepatic failure, and cardiac arrhythmias. Thyroid storm is associated with high mortality (8%-25%), although there has been a paucity of large epidemiologic studies due to rarity of the diagnosis.^1^ Treatment for thyroid storm includes thionamides (propylthiouracil, methimazole, or carbimazole, which is metabolized to methimazole) to inhibit the enzyme thyroid peroxidase in the thyroid, thus reducing the synthesis of triiodothyronine (T_3_) and thyroxine (T_4_). In a previous study, more rapid decline in serum T_3_ levels was seen in patients with hyperthyroidism treated with propylthiouracil compared with methimazole, presumably due to inhibition of peripheral conversion of T_4_ to T_3_.^2^ Thus, the 2016 American Thyroid Association (ATA) guidelines^3^ recommend using propylthiouracil as first-line therapy in the treatment of thyroid storm. On the other hand, the Japan Thyroid Association guidelines recommend using either propylthiouracil or methimazole as first-line therapy in thyroid storm,^4^ based on an observational study of 356 patients showing similar outcomes with methimazole and propylthiouracil.^5^ Large-scale direct comparisons of the effectiveness of propylthiouracil and methimazole in the treatment of thyroid storm are lacking and the optimal first-line thionamide remains unclear. Given the rarity of thyroid storm, it is unlikely that a large-scale randomized clinical trial will be performed to identify the optimal first-line thionamide. Thus, we sought to compare the effectiveness of propylthiouracil with that of methimazole among patients with thyroid storm admitted to US intensive care units (ICUs) or intermediate care units (step-down units).

## Methods

### Cohort

We used the Premier Healthcare Database (January 1, 2016, to December 31, 2020), an enhanced claims-based database that includes time-varying hospital charge data and *International Statistical Classification of Diseases and Related Health Problems, Tenth Revision* (*ICD-10*) codes for approximately 25% of US hospitalizations, to identify patients for inclusion in the study cohort. Included patients were adults (aged ≥18 years) (1) who were admitted to an intermediate care unit or ICU on the first or second day of hospitalization, (2) who had an *ICD-10* code for thyroid storm (E05.01, E05.11, E05.21, E05.31, E05.41, E05.81, or E05.91) designated as present on admission, and (3) who received either propylthiouracil or methimazole (but not both) on the first or second day of hospitalization. To increase the likelihood that the cohort represented thyroid storm rather than severe thyrotoxicosis, we further restricted our cohort to patients who received corticosteroids (hydrocortisone, dexamethasone, or methylprednisolone)—adjunct treatment used during thyroid storm—on the first day of thionamide use. Additional details regarding criteria for study inclusion are included in eTable 1 in Supplement 1. Boston University’s institutional review board waived approval and the requirement for informed consent because this study was designated not human participants research. The protocol for this study was previously deposited in an online repository.^6^ The design of this study was informed by the Strengthening the Reporting of Observational Studies in Epidemiology (STROBE) reporting guideline^7^ and the International Society for Pharmacoeconomics and Outcomes Research (ISPOR) reporting guideline.^8^

### Exposure

The exposure of interest was initiation of propylthiouracil, and the active treatment comparator was initiation of methimazole, ascertained using hospital charge codes. Patients were assigned to the exposure group based on the first thionamide therapy received. Patients who switched therapies on subsequent study days were analyzed based on their initial exposure, akin to an intention-to-treat clinical trial. We assigned study day 0 as the day of thionamide initiation.

### Outcomes

The primary outcome was the composite of in-hospital death or discharge to hospice (as patients discharged to hospice are likely to die shortly after discharge). Secondary outcomes were (1) in-hospital death alone, (2) discharge to hospice alone, (3) organ support–free days by day 21 (a measure of the duration of organ dysfunction accounting for the competing risk of death or discharge to hospice, defined as 21 minus the number of days that a patient received invasive mechanical ventilation or vasopressors after study day 0, or 0 for patients with 21 or more days of organ support or who died during the hospitalization or were discharged to hospice),^9^ (4) costs of thionamide therapy during hospitalization, and (5) total hospital costs. To calculate costs of thionamide therapy, we summed total hospital costs for propylthiouracil or methimazole (medication, equipment, and labor costs) from study day 0 to discharge. To calculate total hospital costs, we summed medication, equipment, and labor costs for all delivered services from study day 0 to hospital discharge. We also quantified potential adverse effects of thionamide therapy using *ICD-10* codes for thyroidectomy, acute hepatic failure, agranulocytosis, and acute pancreatitis that were not present on admission.

### Covariates

Covariates that were likely to confound the association between choice of initial thionamide and the primary composite outcome of death or discharge to hospice were included in models. Covariates captured on hospital admission were age, sex, race and ethnicity (taken from the database), thyroid storm *ICD-10* codes that were designated as primary diagnosis, presence of organ dysfunction (neurologic, cardiovascular, respiratory, hepatic, hematologic, or kidney),^10,11^ severity of comorbidities as defined by Elixhauser comorbidity score,^12,13^ history of congestive heart failure, history of cardiac arrhythmias, and history of liver disease. Covariates captured on study day 0 were use of oral medications, use of vasopressors (separate variables for norepinephrine, epinephrine, vasopressin, dopamine, and phenylephrine), use of β-blockers (separate variables for propranolol, atenolol, metoprolol, and esmolol), type of corticosteroid used, and use of invasive mechanical ventilation. Covariates defined on or before study day 0 were use of iodinated contrast and use of potassium iodide or Lugol iodine solution. Hospital-level covariates were admission to the ICU vs the intermediate care unit, US Census region, hospital bed count, and teaching status. Detailed definitions and codes used to define all study variables (exposure, outcomes, and covariates) are included in eTable 2 in Supplement 1. Missing data occurred in less than 0.01% of variable fields in the Premier Healthcare Database.^14^ No patients included in our study had missing covariate data. For covariates based on diagnostic and procedural codes, patients without codes were interpreted as not having the variable or condition.

### Statistical Analysis

Analyses were conducted from July 2022 to February 2023. Baseline covariates were summarized using counts and percentage for categorical variables and mean (SD) values or median (IQR) values as appropriate, stratified by exposure status. Standardized mean differences (SMDs) were calculated to compare variables by exposure status. We also reported the median (IQR) dose of each thionamide on study day 0 and the number of patient-days evaluated in each exposure group.

We reported the unadjusted absolute risk differences and 95% CIs for the primary outcome and unadjusted difference in mean values for the secondary outcomes. Potential adverse effects were reported as counts and percentages by treatment status.

In the primary adjusted analysis, we used targeted maximum likelihood estimation, a doubly robust, semiparametric, ensemble machine learning analytic approach that yields precise estimates of treatment effects if models for either the exposure or outcome are correctly specified.^15,16,17^ Using targeted maximum likelihood estimation, we reported the adjusted absolute risk difference of the primary outcome for initiation of propylthiouracil compared with initiation of methimazole (ie, negative risk differences mean that exposure to propylthiouracil was associated with lower risk of death or discharge to hospice compared with methimazole). For secondary outcomes, we reported adjusted risk differences (for categorical variables) or adjusted mean differences (for continuous variables). We examined subgroups of (1) use of β-blocker therapy on study day 0, (2) use of potassium iodide or Lugol iodine solution on or before study day 0, and (3) sex. In sensitivity analyses, we (1) restricted to 1 randomly selected hospital admission per patient for patients with multiple admissions, (2) restricted to patients who did not receive glucocorticoids (glucocorticoids inhibit conversion of T_4_ to T_3_ in the periphery; thus, benefits associated with propylthiouracil vs methimazole may be limited to those who do not receive glucocorticoids), and (3) used full optimal matching followed by weighted binomial models with identity link functions and cluster robust SEs^18^ to calculate risk differences.

Analyses were performed with R software, version 4.0.5 (R Group for Statistical Computing). All *P* values were from 2-sided tests, and results were deemed statistically significant at *P* < .05.

## Results

We identified 2845 patients admitted to an ICU or intermediate care unit with a diagnosis of thyroid storm, of whom 1383 patients met full study inclusion criteria (Figure). There were 656 patients (47.4%; mean [SD] age, 45 [16] years; 473 women [72.1%]) who initiated propylthiouracil and 727 (52.6%; mean [SD] age, 45 [16] years; 520 women [71.5%]) who initiated methimazole. The SMD for age was 0.056, and the SMD for sex was 0.013. Baseline covariates stratified by exposure group are shown in Table 1.^19^ Covariates most different between exposure groups were use of potassium iodide or Lugol iodine solution on study day 0 (271 [41.3%] in propylthiouracil group; 189 [26.0%] in methimazole group; SMD, 0.328), type of corticosteroid on study day 0 (hydrocortisone: 564 [86.0%] in propylthiouracil group; 567 [78.0% ] in methimazole group; SMD, 0.209; methylprednisolone: 54 [8.2%] in propylthiouracil group; 115 [15.8%] in methimazole group; SMD, 0.235), and admission to the ICU (480 [73.2%] in propylthiouracil group; 436 [60.0%] in methimazole group; SMD, 0.283). The median total dose was 600 mg (IQR, 300-1000 mg) for propylthiouracil and 40 mg (IQR, 20-60 mg) for methimazole on study day 0.

**Figure.**
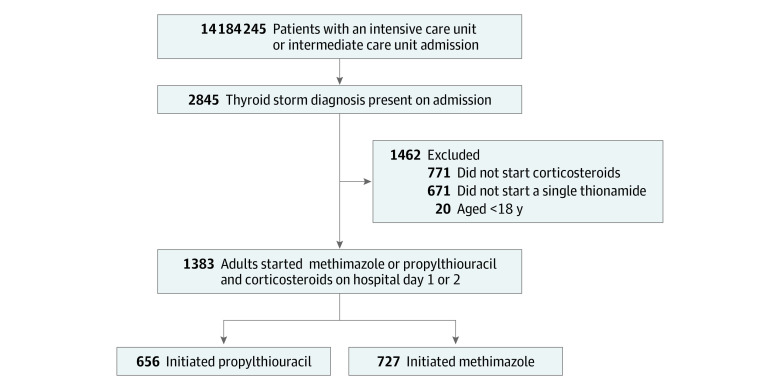
Flow Diagram for Inclusion and Exclusion of Patients

**Table 1.** Baseline Characteristics

Characteristic	No. (%)		Absolute SMD^a^
	Methimazole (n = 727)	Propylthiouracil (n = 656)	
Age, mean (SD), y	45 (16)	45 (16)	0.056
Sex			
Female	520 (71.5)	473 (72.1)	0.013
Male	207 (28.5)	183 (27.9)	
Race			
African American or Black	250 (34.4)	190 (29.0)	0.164
Asian	24 (3.3)	16 (2.4)	
White	377 (51.9)	354 (54.0)	
Other^b^	59 (8.1)	76 (11.6)	
Unknown	17 (2.3)	20 (3.0)	
Ethnicity			
Hispanic or Latinx	74 (10.2)	82 (12.5)	0.075
Not Hispanic or Latinx	506 (69.6)	449 (68.4)	
Unknown	147 (20.2)	125 (19.1)	
Thyroid storm as primary diagnosis	476 (65.5)	440 (67.1)	0.034
Elixhauser comorbidity score, mean (SD)	4 (2)	4 (2)	0.01
History of CHF	276 (38.0)	235 (35.8)	0.044
History of arrhythmia	415 (57.1)	378 (57.6)	0.011
History of liver disease	50 (6.9)	70 (10.7)	0.134
Oral medication delivery other than thionamide on study day 0	680 (93.5)	600 (91.5)	0.079
Potassium iodide or Lugol iodine solution administration on or before study day 0	189 (26.0)	271 (41.3)	0.328
CT with contrast on or before study day 0	134 (18.4)	121 (18.4)	<0.001
Any β-blocker use on study day 0	641 (88.2)	558 (85.1)	0.091
Propranolol	448 (61.6)	392 (59.8)	0.038
Atenolol	21 (2.9)	9 (1.4)	0.105
Metoprolol	251 (34.5)	175 (26.7)	0.171
Esmolol	124 (17.1)	151 (23.0)	0.149
Any vasopressor use on study day 0	72 (9.9)	99 (15.1)	0.157
Norepinephrine	54 (7.4)	68 (10.4)	0.103
Epinephrine	22 (3.0)	35 (5.3)	0.116
Vasopressin	21 (2.9)	37 (5.6)	0.137
Phenylephrine	35 (4.8)	42 (6.4)	0.069
Dopamine	8 (1.1)	18 (2.7)	0.120
Hydrocortisone use on study day 0	567 (78.0)	564 (86.0)	0.209
Dexamethasone use on study day 0	90 (12.4)	80 (12.2)	0.006
Methylprednisolone use on study day 0	115 (15.8)	54 (8.2)	0.235
IMV use on study day 0	102 (14.0)	124 (18.9)	0.132
Acute respiratory organ dysfunction present on admission	83 (11.4)	99 (15.1)	0.109
Acute cardiovascular organ dysfunction present on admission	81 (11.1)	95 (14.5)	0.100
Acute neurologic organ dysfunction present on admission	107 (14.7)	118 (18.0)	0.088
Acute hepatic organ dysfunction present on admission	27 (3.7)	37 (5.6)	0.091
Acute kidney dysfunction present on admission	65 (8.9)	66 (10.1)	0.038
Admission to the ICU (vs step-down unit)	436 (60.0)	480 (73.2)	0.283
Teaching hospital	362 (49.8)	347 (52.9)	0.062
Hospital bed size			
≤99	34 (4.7)	21 (3.2)	0.179
100-199	89 (12.2)	93 (14.2)	
200-299	113 (15.5)	103 (15.7)	
300-399	125 (17.2)	91 (13.9)	
400-499	65 (8.9)	86 (13.1)	
≥500	301 (41.4)	262 (39.9)	
US Census region			
Midwest	115 (15.8)	131 (20.0)	0.139
Northeast	87 (12.0)	78 (11.9)	
South	391 (53.8)	352 (53.7)	
West	134 (18.4)	95 (14.5)	

Abbreviations: CHF, congestive heart failure; CT, computed tomography; ICU, intensive care unit; IMV, invasive mechanical ventilation; SMD, standardized mean difference.

^a^ SMDs less than 0.5 are considered small.^19^

^b^ Provided directly by the Premier Healthcare Database, which did not specify which races were included in that category.

The number of patient-days was 3306 examined in the propylthiouracil group and 3460 in the methimazole group. The primary composite outcome of in-hospital death or hospice discharge occurred in 7.4% (102 of 1383; 95% CI, 6.0%-8.8%) of patients overall, 8.5% (56 of 656; 95% CI, 6.4%-10.7%) of patients in the propylthiouracil group, and 6.3% (46 of 727; 95% CI, 4.6%-8.1%) in the methimazole group (Table 2). The adjusted risk difference was 0.6% (95% CI, −1.8% to 3.0%) (*P* = .64). There were also no differences in in-hospital death alone (adjusted risk difference, 1.1% [95% CI, −1.2% to 3.5%]; *P* = .34), discharge to hospice alone (adjusted risk difference, −0.6% [95% CI, −1.6% to 0.3%]; *P* = .20), organ support–free days (propylthiouracil-methimazole–adjusted mean difference, −0.2 days [95% CI, −0.7 to 0.3 days]; *P* = .40), or total costs of hospitalization (propylthiouracil-methimazole–adjusted mean difference, $1097 [95% CI, −$972 to $3166]; *P* = .30]). Total costs of thionamide treatment were higher for the propylthiouracil group compared with the methimazole group (adjusted mean difference, $72 [95% CI, $48 to $97]; *P* < .001). Adverse events, including treatment failure leading to thyroidectomy, hepatic failure, agranulocytosis, and pancreatitis, were low for both treatment groups (Table 3).

**Table 2.** Comparison of Outcomes Between Methimazole-Treated and Propylthiouracil-Treated Patients With Thyroid Storm

Outcome	Methimazole (n = 727)	Propylthiouracil (n = 656)	Difference		*P* value
			Unadjusted	Adjusted	
Composite of in-hospital death or discharge to hospice (95% CI), %	6.3 (4.6 to 8.1)	8.5 (6.4 to 10.7)	2.2 (−0.6 to 5.0)	0.6 (−1.8 to 3.0)	.64
In-hospital death (95% CI), %	5.0 (3.4 to 6.5)	7.9 (5.9 to 10.0)	3.0 (0.4 to 5.6)	1.1 (−1.2 to 3.5)	.34
Discharge to hospice (95% CI), %	1.4 (0.5 to 2.2)	0.6 (0.0 to 1.2)	−0.8 (−1.8 to 0.3)	−0.6 (−1.6 to 0.3)	.20
Organ support–free days, mean (95% CI)	19.0 (18.6 to 19.4)	18.2 (17.7 to 18.7)	−0.8 (−1.4 to −0.2)	−0.2 (−0.7 to 0.3)	.40
Total cost of thionamide, mean (95% CI), $	58 (51 to 65)	143 (115 to 172)	85 (57 to 113)	72 (48 to 97)	<.001
Total cost of hospitalization, mean (95% CI), $	18 068 (16 497 to 19 640)	21 474 (18 973 to 23 976)	3406 (513 to 6299)	1097 (−972 to 3166)	.30

**Table 3.** Adverse Events in Methimazole-Treated and Propylthiouracil-Treated Patients With Thyroid Storm

Adverse event	Methimazole (n = 727)	Propylthiouracil (n = 656)
Thyroidectomy	1	2
Acute hepatic failure secondary to toxin or drug	0	1
Agranulocytosis	1	1
Acute pancreatitis	0	0

Subgroup analyses, similar to the primary analysis, showed no differences in the primary outcome between exposure groups (eTable 3 in Supplement 1). Sensitivity analyses limiting to 1 randomly selected hospital admission per patient (n = 1341; adjusted risk difference, 0.4% [95% CI, −2.0% to 2.8%]; *P* = .75), limiting to patients who did not receive glucocorticoids (n = 752; adjusted risk difference, −1.3% [95% CI, −3.7% to 1.2%]; *P* = .31), and using full optimal matching (eFigure in Supplement 1) (n = 2845; adjusted risk difference, −0.6% [95% CI, −3.7% to 2.4%]; *P* = .68) were similar to the primary targeted maximum likelihood estimation analysis.

## Discussion

We compared outcomes associated with use of propylthiouracil with those associated with use of methimazole to treat thyroid storm using a large, multicenter, US-based database. We found no differences in the composite outcome of in-hospital mortality or discharge to hospice, in-hospital mortality alone, duration of organ support, or total hospitalization costs and only a small decrease in thionamide-specific costs. These results suggest that propylthiouracil and methimazole can be used interchangeably in the management of thyroid storm.

Our results should be considered in the context of prior studies. Two Japan-based studies^5,20^ (n = 1324 and n = 356) examined risk factors for mortality among patients with thyroid storm. In both studies, methimazole use (85% and 66%) was more common than propylthiouracil use, but thionamide choice was not associated with mortality in univariate analyses. Although these studies also used large multicenter claims-based cohorts, there are several key differences between these prior studies and our own. In contrast to these studies, we found more balanced use of propylthiouracil and methimazole in a US-based multicenter cohort, which may reflect US-based ATA guidelines^3^ that recommend using propylthiouracil as first-line therapy. Others have speculated that the preferential use of methimazole in Japan may be due to methimazole being more readily available in intravenous formulation in Japan compared with propylthiouracil,^5^ as intravenous administration would be preferred for critically ill patients with impaired consciousness or severe gastrointestinal symptoms. Another key difference between prior studies and our own is that our study was designed specifically to identify the comparative effectiveness of propylthiouracil with that of methimazole, using robust target trial causal frameworks and multivariable analyses, thus providing robust evidence that there are no clinically significant differences in patient outcomes when using methimazole vs propylthiouracil. Overall mortality in our cohort was 7.4%, similar to studies from Taiwan (8.1%)^21^ and Germany (7.4%),^22^ but higher than the 1.2% to 3.6% per year between 2004 and 2013 reported in a previous US study.^23^ We speculate that the higher mortality in our study compared with prior US-based studies is due to our requirement for patients to have received both corticosteroids and thionamides, not just a claims-based diagnosis for thyroid storm.

### Strengths and Limitations

Our study has several strengths. To our knowledge, this study is the largest to date comparing mortality with different thionamide treatments among patients with thyroid storm, as well as the largest study assessing mortality outcomes of patients with thyroid storm. In addition, we used advanced modeling techniques to increase estimate precision and a robust target trial framework that included the use of an active comparator and a specific definition of study time 0, which helped reduce selection and immortal time bias.^24^

Our study also has some limitations. To our knowledge, there are currently no standard criteria for diagnosis of thyroid storm, although the Burch-Wartofsky criteria^25^ and criteria outlined by Akamizu and colleagues^26^ are generally used. In addition, thyroid storm is a clinical diagnosis, which may be subject to the assessment by the treating clinicians. Our use of a claims-based thyroid storm diagnosis is consistent with approaches used by others when using large multicenter cohorts,^20,23^ but it has not been validated compared with existing clinical scoring systems. However, given that claims-based definitions are generally insensitive but highly specific^27^ and that we required patients with a thyroid storm diagnosis present on admission to receive both a thionamide and corticosteroid, it is likely that patients in our cohort did have thyroid storm. We did not have access to medications patients were taking prior to hospitalization. Thus, it is possible that some patients labeled as initiating methimazole or propylthiouracil on hospital day 1 or 2 were instead continuing a home medication. Moreover, we were unable to ascertain the reason for choosing a certain thionamide as the initial treatment option. Methimazole may be preferred over propylthiouracil given propylthiouracil’s association with rare cases of fulminant hepatic failure and more severe cases of liver disease.^28^ However, we did not see significant differences in complications from the medications between the 2 groups, with only 1 case of hepatic failure associated with propylthiouracil (Table 3). We limited enrollment to patients with thyroid storm present on admission and who received methimazole or propylthiouracil within 2 days of hospitalization to minimize confounding associated with time such as immortal time bias. However, given that the cohort included only patients with recognized thyroid storm that was treated early in the hospital course, whether results would be similar among patients with delayed diagnosis or treatment is unclear. In addition, we emulated an intention-to-treat clinical trial focused on comparing outcomes between initiation of propylthiouracil vs methimazole, not subsequent titration of either of these medications. Thus, our results should not be generalized to clinical scenarios involving subsequent thionamide doses. Last, the median total dose of methimazole on study day 0 was 40 mg (IQR, 20-60 mg), lower than the dose of 60 to 80 mg recommended by the ATA.^3^ The lower-than-expected dose of methimazole could represent routine clinical practice that diverges from guidelines, undertreatment of thyroid storm in patients who received methimazole, or that patients who received methimazole (prior to model adjustment) had less-severe thyroid storm.

## Conclusions

Using target trial emulation and a large, US-based multicenter claims-based database, this comparative effectiveness study found no significant differences in clinical outcomes or adverse effects between methimazole and propylthiouracil in the management of thyroid storm. These results suggest that propylthiouracil and methimazole can be used interchangeably in the management of thyroid storm. Given the findings of this study, as well as other studies, current ATA recommendations on the choice of thionamides may benefit from reevaluation.

## Notes

Supplement 1. **eTable 1.** Study Inclusion Criteria **eTable 2.** Study Variables **eTable 3.** Subgroup Analyses of Adjusted Risk Differences of Mortality Between Methimazole-Treated and Propylthiouracil-Treated Patients With Thyroid Storm **eFigure.** Love Plot Showing Change in Standardized Mean Differences Before and After Full Optimal Matching

Supplement 2.
Data Sharing Statement
