# Supplementary material for: Comparison of Propylthiouracil vs Methimazole for Thyroid Storm in Critically Ill Patients
Source: JAMA Netw Open. 2023 Apr 17;6(4):e238655. doi: 10.1001/jamanetworkopen.2023.8655 (PMC10111182; doi:10.1001/jamanetworkopen.2023.8655)
Supplement: Supplement 1. — eTable 1. Study Inclusion Criteria eTable 2. Study Variables eTable 3. Subgroup Analyses of Adjusted Risk Differences of Mortality Between Methimazole-Treated and Propylthiouracil-Treated Patients With Thyroid Storm eFigure. Love Plot Showing Change in Standardized Mean Differences Before and After Full Optimal Matching [file jamanetwopen-e238655-s001.pdf]

Supplementary Online Content

Lee SY, Modzelewski KL, Law AC, Walkey AJ, Pearce EN, Bosch NA. Comparison of propylthiouracil vs methimazole for thyroid storm in critically ill patients. *JAMA Netw Open.* 2023;6(4):e238655. doi:10.1001/jamanetworkopen.2023.8655

eTable 1. Study Inclusion Criteria
eTable 2. Study Variables
eTable 3. Subgroup Analyses of Adjusted Risk Differences of Mortality Between Methimazole-Treated and Propylthiouracil-Treated Patients With Thyroid Storm
eFigure. Love Plot Showing Change in Standardized Mean Differences Before and After Full Optimal Matching

This supplementary material has been provided by the authors to give readers additional information about their work.

© 2023 Lee SY et al. *JAMA Network Open.*

eTable 1. Study Inclusion Criteria

Variable	Definition
Adults	From the PATDEMO table, AGE (age in years on admission) ≥18
Admission to an intermediate care unit or intensive care unit on the first or second day of hospitalization	From the PATBILL table, SERV_DAY (hospital day) equal to 1 or 2 and STD_CHG_CODE (charge codes) equal to any of the following:<br><br>110101000050000 110101000130000 110110000380000 110120000390000 110140000520000 110150000010000 110164000580000 110173000030000 110173000190000 110174000050000 110174000190000 110200000090000 110200000190000 110200000420000 110201000080000 110201000260000 110202000070000 110202000120000 110203000210000 110203000440000 110204000860000 110204000870000 110204000880000 110204000890000 110204000900000 110204000910000 110206000010000 110206000030000 110206000040000 110206000050000 110206000052000 110206000090000 110206000150000 110206000190000 110206000300000 110206000310000 110206000330000 110206000500000 110206000510000 110209000910000 110210000010000 110210000020000 110210000030000 110210000060000 110210000190000 110214000010000 110214000020000 110214000030000 110214000100000 110214000140000 110214000480000 110214000490000 110214000620000
Thyroid storm diagnosis	From the PATICD_DIAG table, ICD_POA (present on admission identifier) equal to “Y” and ICD_CODE (International classification of diseases, tenth revision code) for any of the following:<br><br>E05.01 E05.11 E05.21 E05.31 E05.41 E05.81 E05.91
Received propylthiouracil or methimazole on the first or second hospital day	From the PATBILL table, SERV_DAY equal to 1 or 2 and STD_CHG_CODE equal to any of the following:<br><br>250250041520000 250250041530000 250250056060000 250888013390000 250888017920000 250999011180000 250999015080000
Received corticosteroids on the first day of thionamide use	From the PATBILL table, SERV_DAY equal to the first SERV_DAY that patient met criteria for receiving propylthiouracil or methimazole (study day 0) and STD_CHG_CODE equal to any of the following:<br><br>250250017060000 250250017070000 250250017080000 250250017090000 250250017100000 250250017110000 250250017120000 250250017130000 250250017140000 250250017150000 250250017210000 250250017220000 250250017230000 250250017240000 250250017250000 250250017260000 250250017270000 250250017280000

© 2023 Lee SY et al. *JAMA Network Open.*

	250250017290000 250250017300000 250250017310000 250250017320000 250250017330000 250250017340000 250250017360000 250250017370000 250250017380000 250250017390000 250250017400000 250250017410000 250250017420000 250250017580000 250250031510000 250250031520000 250250031530000 250250031540000 250250031550000 250250031560000 250250031570000 250250031580000 250250031590000 250250031750000 250250032030000 250250032040000 250250032050000 250250042240000 250250042250000 250250042260000 250250042270000 250250042280000 250250042290000 250250042300000 250250042310000 250250042320000 250250042330000 250250042340000 250250042350000 250250118750000 250250120700000 250888005850000 250888005860000 250888010270000 250888010280000 250888010290000 250888013670000 250888013680000

© 2023 Lee SY et al. *JAMA Network Open.*

eTable 2. Study Variables

Variable	Definition
Exposures	
Propylthiouracil	From the PATBILL table, SERV_DAY equal to 1 or 2 and STD_CHG_CODE equal to 250250056060000 250888017920000 or 250999015080000 and STD_CHG_CODE not equal to 250250041520000 250250041530000 250888013390000 or 250999011180000 on the SERV_DAY where the criteria for propylthiouracil were first met (study day 0)
Methimazole	From the PATBILL table, SERV_DAY equal to 1 or 2 and STD_CHG_CODE equal to 250250041520000 250250041530000 250888013390000 or 250999011180000 and STD_CHG_CODE not equal to 250250056060000 250888017920000 or 250999015080000 on the SERV_DAY where the criteria for methimazole were first met (study day 0)
Outcomes	
Hospital death or discharge to hospice	From the PATDEMO table, DISC_STATUS (discharge status) equal to 20, 50, or 51
Organ support free days by day 21	An organ support day was defined as any SERV_DAY from the PATBILL table where STD_CHG_CODEs were present for vasopressors (250250047730000 250250047740000 250250131530000 250250131660000 250250131670000 250888015040000 250250023270000 250250023280000 250250023290000 250250023300000 250250023310000 250250023320000 250250023400000 250250023410000 250250023420000 250250118440000 250888007600000 250250066000000 250250066010000 250250066020000 250250066030000 250888022100000 250250051420000 250250051430000 250250131740000 250250131780000 250250131790000 250250131800000 250888016620000 250250020350000 250250020360000 250250020370000 250250020380000 250250020390000 250250020400000 250250020410000 250250020420000 250250020430000 250250020440000 250250020450000 250250020460000 250250020470000 250250020480000 250250020490000 250250020500000 250250117320000 250888006920000) or invasive mechanical ventilation

© 2023 Lee SY et al. *JAMA Network Open.*

	(270270013950000 270270057050000 270270086600000 270270088900000 270270089300000 290290093620000 410412946560000 410412946560001 410412946570000 410412946570004 410412946570005 410412946570007 410412946570009 970976946560000 970976946570000)<br><br>The number of organ support days was calculated by summing the number of organ support days per patient starting with study day 1 (the day after first thionamide use)<br><br>When hospital death or discharge to hospice was true or when hospital death or discharge to hospice was false and the number of organ support days was ≥21, organ support free days by day 21 was set to 0. When hospital death or discharge to hospice was false and the number of organ support days was <21, organ support free days by day 21 was defined using the following equation: 21 – organ support days.
Cost of thionamide therapy during hospitalization	From the PATBILL table, the sum of BILL_COST where STD_CHG_CODE was equal to any of the following:<br><br>250250041520000 250250041530000 250250056060000 250888013390000 250888017920000 250999011180000 250999015080000
Total hospital costs	From the PATBILL table, the sum of BILL_COST
Potential adverse effects not present on admission	
Thyroidectomy	From the PATICD_PROC table, where ICD_CODE equaled 0GBG 0GBG0 0GBG0Z 0GBG0ZX 0GBG0ZZ 0GBG3 0GBG3Z 0GBG3ZX 0GBG3ZZ 0GBG4 0GBG4Z 0GBG4ZX 0GBG4ZZ 0GBH 0GBH0 0GBH0Z 0GBH0ZX 0GBH0ZZ 0GBH3 0GBH3Z 0GBH3ZX 0GBH3ZZ 0GBH4 0GBH4Z 0GBH4ZX 0GBH4ZZ 0GBJ 0GBJ0 0GBJ0Z 0GBJ0ZX 0GBJ0ZZ 0GBJ3 0GBJ3Z 0GBJ3ZX 0GBJ3ZZ 0GBJ4 0GBJ4Z 0GBJ4ZX or 0GBJ4ZZ and where PROC_DAY was greater than study day 0
Acute hepatic failure	From the PATICD_DIAG table, where ICD_CODE equaled K71.0 K71.1 K71.10 K71.11 K71.2 K71.3 K71.4 K71.5 K71.50 K71.51 K71.6 K71.7 K71.8 or K71.9 and where ICD_POA equaled “Y”

© 2023 Lee SY et al. *JAMA Network Open.*

Agranulocytosis	From the PATICD_DIAG table, where ICD_CODE equaled D70.2 and where ICD_POA equaled “Y”
Acute pancreatitis	From the PATICD_DIAG table, where ICD_CODE equaled K85.0 K85.00 K85.01 K85.02 K85.1 K85.10 K85.11 K85.12 K85.2 K85.20 K85.21 K85.22 K85.3 K85.30 K85.31 K85.32 K85.8 K85.80 K85.81 K85.82 K85.9 K85.90 K85.91 or K85.92 and where ICD_POA equaled “Y”
Covariables	
Age	From the PATDEMO table, AGE variable
Sex	From the PATDEMO table, database defined categories of Male, Female, and Unknown. Note: There were no instances of Unknown Sex included in the cohort.
Race[a]	From the PATDEMO table, database defined categories of African American/Black, Asian, White, Other, and Unknown.
Ethnicity[b]	From the PATDEMO table, database defined categories of Hispanic, Not Hispanic, and Unknown.
Primary diagnosis of thyroid storm	From the PATICD_DIAG table, ICD_POA (present on admission identifier) equal to “Y”, ICD_PRI_SEC (primary/secondary identifier) equal to “P” and ICD_CODE (International classification of diseases, tenth revision code) for any of the following:<br><br>E05.01 E05.11 E05.21 E05.31 E05.41 E05.81 E05.91
Neurologic organ dysfunction	From the PATICD_DIAG table, ICD_POA (present on admission identifier) equal to “Y” and ICD_CODE (International classification of diseases, tenth revision code) for any of the following:<br><br>G93.40 G93.41 G93.49 I67.83 F05 F06.2 F06.0 F06.30 F06.31 F06.32 F06.33 F06.34 F06.4 F06.1 F53 F06.8 G93.1

© 2023 Lee SY et al. *JAMA Network Open.*

Cardiovascular organ dysfunction	From the PATICD_DIAG table, ICD_POA (present on admission identifier) equal to "Y" and ICD_CODE (International classification of diseases, tenth revision code) for any of the following:<br><br>R57.9 R57.0 R65.21 R57.1 R57.8 I95.1 I95.2 I95.3 I95.81 I95.89 I95.9
Respiratory organ dysfunction	From the PATICD_PROC table, PROC_DAY (day of procedure) equal to 1 and ICD_CODE (International classification of diseases, tenth revision code) for any of the following:<br><br>5A1935Z 5A1945Z 5A1955Z
Hepatic organ dysfunction	From the PATICD_DIAG table, ICD_POA (present on admission identifier) equal to "Y" and ICD_CODE (International classification of diseases, tenth revision code) for any of the following:<br><br>K72.00 K72.01 K76.2 K76.3
Hematologic organ dysfunction	From the PATICD_DIAG table, ICD_POA (present on admission identifier) equal to "Y" and ICD_CODE (International classification of diseases, tenth revision code) for any of the following:<br><br>D69.59 D69.51 D69.6 D65 D68.8 D68.9
Renal organ dysfunction	From the PATICD_DIAG table, ICD_POA (present on admission identifier) equal to "Y" and ICD_CODE (International classification of diseases, tenth revision code) for any of the following:<br><br>N17.0 N17.1 N17.2 N17.8 N17.9
Elixhauser comorbidity score	From the PATICD_DIAG table, ICD_POA (present on admission identifier) equal to "Y" and ICD_CODE then inputted into 'comorbidity' function from the comorbidity R package using the following syntax: "comorbidity(code="ICD_CODE", id="PAT_KEY", score="elixhauser", assign0=T, icd="icd10", tidy.codes=T)". From this function, the "score" output was defined as the Elixhauser comorbidity score

© 2023 Lee SY et al. *JAMA Network Open.*

History of congestive heart failure	The “chf” output from the comorbidity function outlined in the elixhauser cormorbidity score definition
History of cardiac arrhythmias	The “carit” output from the comorbidity function outlined in the elixhauser cormorbidity score definition
History of liver disease	The “ld” output from the comorbidity function outlined in the elixhauser cormorbidity score definition
per OS medication use	From the PATBILL table, SERV_DAY equal study day 0 and any STD_CHG_CODE linked to a STD_CHG_CODE in the CHGMSTR table where PROD_NAME_METH_DESC contained the word “ORAL” and SUM_DEPT_DESC equaled “PHARMACY” and the STD_CHG_CODE was not a code for methimazole or propylthiouracil
Use of norepinephrine	From the PATBILL table, SERV_DAY equal study day 0 and any STD_CHG_CODE linked to a STD_CHG_CODE in the CHGMSTR table where PROD_NAME_METH_DESC equaled “NOREPINEPHRINE PARENTERAL”
Use of epinephrine	From the PATBILL table, SERV_DAY equal study day 0 and any STD_CHG_CODE linked to a STD_CHG_CODE in the CHGMSTR table where PROD_NAME_METH_DESC equaled “EPINEPHRINE PARENTERAL” and STD_CHG_DESC did not equal "EPINEPHRINE, EPIPEN INJ 0.3MG", "EPINEPHRINE, EPIPEN JR INJ 0.15MG"
Use of vasopressin	From the PATBILL table, SERV_DAY equal study day 0 and any STD_CHG_CODE linked to a STD_CHG_CODE in the CHGMSTR table where PROD_NAME_METH_DESC equaled “VASOPRESSIN PARENTERAL”
Use of dopamine	From the PATBILL table, SERV_DAY equal study day 0 and any STD_CHG_CODE linked to a STD_CHG_CODE in the CHGMSTR table where PROD_NAME_METH_DESC equaled “DOPAMINE PARENTERAL”
Use of phenylephrine	From the PATBILL table, SERV_DAY equal study day 0 and any STD_CHG_CODE linked to a STD_CHG_CODE in the CHGMSTR table where PROD_NAME_METH_DESC equaled “PHENYLEPH PARENTERAL”

© 2023 Lee SY et al. *JAMA Network Open.*

Use of propranolol	From the PATBILL table, SERV_DAY equal study day 0 and any STD_CHG_CODE linked to a STD_CHG_CODE in the CHGMSTR table where PROD_NAME_METH_DESC equaled “PROPRANOLOL ORAL” or “PROPRANOLOL PARENTERAL”
Use of atenolol	From the PATBILL table, SERV_DAY equal study day 0 and any STD_CHG_CODE linked to a STD_CHG_CODE in the CHGMSTR table where PROD_NAME_METH_DESC equaled “ATENOLOL ORAL” or “ATENOLOL PARENTERAL”
Use of metoprolol	From the PATBILL table, SERV_DAY equal study day 0 and any STD_CHG_CODE linked to a STD_CHG_CODE in the CHGMSTR table where PROD_NAME_METH_DESC equaled “METOPROLOL ORAL” or “METOPROLOL PARENTERAL”
Use of esmolol	From the PATBILL table, SERV_DAY equal study day 0 and any STD_CHG_CODE linked to a STD_CHG_CODE in the CHGMSTR table where PROD_NAME_METH_DESC equaled “ESMOLOL PARENTERAL”
Type of corticosteroid used (hydrocortisone)	From the PATBILL table, SERV_DAY equal to the first SERV_DAY that patient met criteria for receiving propylthiouracil or methimazole (study day 0) and STD_CHG_CODE equal to any of the following:<br><br>250250031540000 250250031550000 250250031560000 250250031570000 250250031580000 250250031590000 250888010270000 250888010280000
Type of corticosteroid used (dexamethasone)	From the PATBILL table, SERV_DAY equal to the first SERV_DAY that patient met criteria for receiving propylthiouracil or methimazole (study day 0) and STD_CHG_CODE equal to any of the following:<br><br>250250017060000 250250017070000 250250017080000 250250017090000 250250017100000 250250017110000 250250017120000 250250017130000 250250017140000 250250017150000 250250017210000 250250017220000 250250017230000 250250017240000 250250017250000 250250017260000 250250017270000 250250017280000 250250017290000 250250017300000 250250017310000 250250017320000 250250017330000 250250017340000

© 2023 Lee SY et al. *JAMA Network Open.*

	250250017360000 250250017370000 250250017380000 250250017390000 250250017400000 250250017410000 250250017420000 250250017580000 250250118750000 250250120700000 250888005850000 250888005860000
Type of corticosteroid used (methylprednisolone)	From the PATBILL table, SERV_DAY equal to the first SERV_DAY that patient met criteria for receiving propylthiouracil or methimazole (study day 0) and STD_CHG_CODE equal to any of the following:<br><br>250250042240000 250250042250000 250250042260000 250250042270000 250250042280000 250250042290000 250250042300000 250250042310000 250250042320000 250250042330000 250250042340000 250250042350000 250888013670000 250888013680000
Use of invasive mechanical ventilation	From the PATBILL table, SERV_DAY equal to the first SERV_DAY that patient met criteria for receiving propylthiouracil or methimazole (study day 0) and STD_CHG_CODE equal to any of the following:<br><br>270270013950000 270270057050000 270270086600000 270270088900000 270270089300000 290290093620000 410412946560000 410412946560001 410412946570000 410412946570004 410412946570005 410412946570007 410412946570009 970976946560000 970976946570000
Use of ionic contrast	From the PATBILL table, SERV_DAY equal study day 0 and any STD_CHG_CODE linked to a STD_CHG_CODE in the CHGMSTR table where STD_DEPT_DESC is equal to “RADIOLOGY” and CLIN_DTL_DESC contains the string “W/ CONTRAST and the string “CT”
Use of potassium iodide or Lugol’s solution	From the PATBILL table, SERV_DAY equal study day 0 and any STD_CHG_CODE linked to a STD_CHG_CODE in the CHGMSTR table where STD_CHG_DESC is equal to any of the following:<br><br>"POT IOD, SSKI SOL 1GM/ML 15ML", "POT IOD, SSKI SOL 1GM/ML 1ML", "POT IOD, SSKI SOL 1GM/ML 30ML", "IODINE(STRONG), LUGOLS SOLN 120ML", "IODINE(STRONG), LUGOLS SOLN 15ML", "IODINE(STRONG), LUGOLS SOLN

© 2023 Lee SY et al. *JAMA Network Open.*

	30ML", "IODINE(STRONG), LUGOLS SOLN 480ML", "IODINE(STRONG), LUGOLS SOLN 5ML"
Admission to the intensive care unit (versus intermediate care unit)	From the PATBILL table, SERV_DAY (hospital day) equal to 1 or 2 and STD_CHG_CODE (charge codes) equal to any of the following:<br><br>110173000030000 110173000190000 110174000050000 110174000190000 110200000090000 110200000190000 110200000420000 110201000080000 110201000260000 110202000070000 110202000120000 110203000210000 110203000440000 110204000860000 110204000870000 110204000880000 110204000890000 110204000900000 110204000910000 110209000910000 110210000010000 110210000020000 110210000030000 110210000060000 110210000190000
Hospital United States census region	From the PROVIDERS table, the PROV_REGION variable
Hospital bed count	From the PROVIDERS table, the BEDS_GRP variable
Hospital teaching status	From the PROVIDERS table, the TEACHING variable

[a]Race, similar to other covariables, was included as a model covariable given this variable could potentially confound the relationship between exposure status and outcomes. Race was dichotomized in models as white race yes/no

[b]Ethnicity was included in Table 1 as a baseline characteristic

© 2023 Lee SY et al. *JAMA Network Open.*

eTable 3. Subgroup Analyses of Adjusted Risk Differences of Mortality Between Methimazole-Treated and Propylthiouracil-Treated Patients With Thyroid Storm

**Subgroup**	**Adjusted risk difference for propylthiouracil compared to methimazole (95% CI)**
Beta-blocker use	
Yes (n=1199)	0.2 (-2.4, 2.8)
No (n=184)	-0.3 (-7.0, 6.3)
SSKI/Lugol's use	
Yes (n=460)	-0.7 (-4.6, 3.3)
No (n=923)	0.9 (-2.0, 3.8)
Sex	
Female (n=993)	0.7 (-2.9, 3.5)
Male (n=390)	-0.8 (-4.6, 3.1)

© 2023 Lee SY et al. *JAMA Network Open.*

eFigure. Love Plot Showing Change in Standardized Mean Differences Before and After Full Optimal Matching

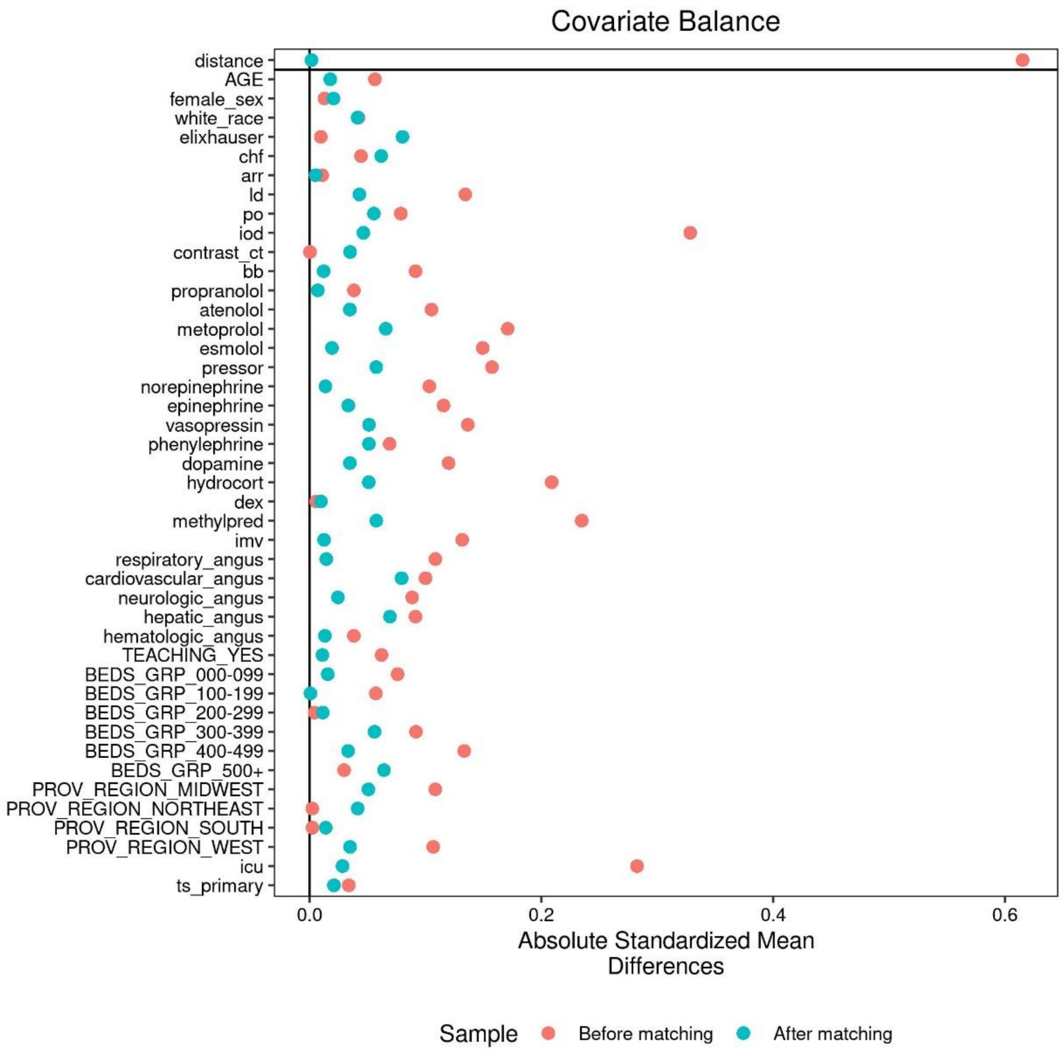

© 2023 Lee SY et al. *JAMA Network Open.*
