# Supplementary material for: Comparison of Propylthiouracil vs Methimazole for Thyroid Storm in Critically Ill Patients
Source: JAMA Netw Open. 2023 Apr 17;6(4):e238655. doi: 10.1001/jamanetworkopen.2023.8655 (PMC10111182; doi:10.1001/jamanetworkopen.2023.8655)
Supplement: Supplement 2. — Data Sharing Statement [file jamanetwopen-e238655-s002.pdf]

Data Sharing Statement

Lee. Comparison of Propylthiouracil vs Methimazole for Thyroid Storm in Critically Ill Patients. *JAMA Netw Open*. Published April 17, 2023. doi:10.1001/jamanetworkopen.2023.8655

Data
Data available: No

Additional Information
Explanation for why data not available: The Premier Healthcare Database is the property of Premier, Inc. and is used by the investigators as part of a data use agreement that does not allow the data to be shared outside of the investigators institution.
